# Mitochondrial division inhibitor (mdivi-1) induces extracellular matrix (ECM)-detachment of viable breast cancer cells by a DRP1-independent mechanism

**DOI:** 10.1038/s41598-024-64228-9

**Published:** 2024-06-19

**Authors:** Eduardo Silva-Pavez, Elizabeth Mendoza, Pablo Morgado-Cáceres, Ulises Ahumada-Castro, Galdo Bustos, Matías Kangme-Encalada, Amaia Lopez de Arbina, Andrea Puebla-Huerta, Felipe Muñoz, Lucas Cereceda, Manuel Varas-Godoy, Yessia Hidalgo, J. Cesar Cardenas

**Affiliations:** 1 Facultad de Odontología y Ciencias de la Rehabilitación, Universidad San Sebastián, Bellavista, Bellavista 7, Recoleta, Santiago Chile; 2 Center for Integrative Biology, Faculty of Sciences, Universidad Mayor, Camino la Pirámide 5750, Huechuraba, Santiago Chile; 3 Geroscience Center for Brain Health and Metabolism, Santiago, Chile; 4 IMPACT, Center of Interventional Medicine for Precision and Advanced Cellular Therapy, Santiago, Chile; 5 Laboratory of Nano-Regenerative Medicine, Biomedical Research and Innovation Center (CIIB), Faculty of Medicine, Universidad de los Andes, Santiago, Chile; 6 Biodonostia Health Research Institute, 20014 San Sebastián, Spain; 7 Cancer Cell Biology Lab., Centro de Biología Celular y Biomedicina (CEBICEM), Facultad de Medicina y Ciencia, Universidad San Sebastián, Lota 2465, Santiago, Chile; 8 Centro Ciencia & Vida, Fundación Ciencia & Vida, Avenida Del Valle Norte 725, Huechuraba, Santiago Chile; 9 Advanced Center for Chronic Diseases (ACCDiS), Faculty of Medicine, Universidad de Chile, Santos Dumont 964, Independencia, Santiago Chile; 10 Buck Institute for Research on Aging, Novato, USA; 11 Department of Chemistry and Biochemistry, University of California, Santa Barbara, USA

**Keywords:** Mdivi-1, Cell detachment, Mitochondrial complex I, Cancer, Metabolism, Biochemistry, Cancer, Cell biology

## Abstract

Increasing evidence supports the hypothesis that cancer progression is under mitochondrial control. Mitochondrial fission plays a pivotal role in the maintenance of cancer cell homeostasis. The inhibition of DRP1, the main regulator of mitochondrial fission, with the mitochondrial division inhibitor (mdivi-1) had been associated with cancer cell sensitivity to chemotherapeutics and decrease proliferation. Here, using breast cancer cells we find that mdivi-1 induces the detachment of the cells, leading to a bulk of floating cells that conserved their viability. Despite a decrease in their proliferative and clonogenic capabilities, these floating cells maintain the capacity to re-adhere upon re-seeding and retain their migratory and invasive potential. Interestingly, the cell detachment induced by mdivi-1 is independent of DRP1 but relies on inhibition of mitochondrial complex I. Furthermore, mdivi-1 induces cell detachment rely on glucose and the pentose phosphate pathway. Our data evidence a novel DRP1-independent effect of mdivi-1 in the attachment of cancer cells. The generation of floating viable cells restricts the use of mdivi-1 as a therapeutic agent and demonstrates that mdivi-1 effect on cancer cells are more complex than anticipated.

## Introduction

Increasing evidence suggests that mitochondria play a pivotal role in maintaining cancer cell homeostasis by regulating mitochondrial metabolism in response to energetic demands^1,2^. A reduction in mitochondrial respiration and oxidative phosphorylation (OXPHOS) has been shown to induce cell death in lymphoblastic leukemia cells^3^ in glioblastoma^4^, in starvation-resistant renal cell carcinomas^5^, pancreatic cancer^6^ and breast cancer cells^7^. Even in OXPHOS-defective cancer cells, mitochondrial function remains essential, providing the building block that sustains reductive carboxylation and cellular homeostasis^8^. Therefore, it is unsurprising that metastasis, which results from a multi-step process known as the metastatic cascade, is also influenced by mitochondrial-regulated signaling^9^.

During the early stages of metastasis, cancer cells must detach from the primary tumor’s extracellular matrix (ECM). This detachment enables the cancer cells to spread to other organs through the circulatory or lymphatic systems^10^. These detached cells, known as circulating tumor cells (CTCs), play a significant role in metastasis formation^11^. Indeed, the number of CTCs has been identified as an independent predictor of progression-free survival and overall survival in patients with metastatic breast cancer^12^. Nevertheless, the mechanisms underlying the loss of cell adhesion to the ECM, particularly during cancer cell detachment from the primary tumor, remain poorly understood.

Mitochondrial complex I, the largest respiratory complex of the electron transport chain (ETC) and critical for OXPHOS^13^, as well as for mitochondrial calcium (Ca^2+^) homeostasis^14^, has been shown to enhance cancer aggressiveness and metastasis when specific subunits such as NDUFV1, NDUFA13, NDUFS3 or NDUFB9 are down-regulated or when its activity is inhibited by small molecules^13^. In these scenarios, reactive oxygen species (ROS) play a significant role^15^.

Mdivi-1 is a small molecule from the quinazolinone family that inhibits the activity of dynamin-related protein 1 (DRP1) and, as a result, inhibits mitochondrial fission. This molecule has shown great potential as a therapeutic agent in cancer treatment. Studies have shown that mdivi-1 can decrease proliferation in thyroid and lung cancer cells^16,17^ and induce apoptosis in pancreatic stem cancer cells^18^. However, recent research has identified complex I as a new target of mdivi-1^19^. These findings suggest that we must re-examine our understanding of mdivi-1’s effects on cancer cell biology.

Our data suggests that mdivi-1 reduce cell proliferation and induces the detachment of viable breast cancer cells that maintain their proliferative, migratory, and invasive potential upon re-seeding. This effect of mdivi-1 depends on the inhibition of active complex I and is independent of DRP1. Finally, we have determined that the pentose phosphate pathway (PPP) plays a central role in promoting cell detachment after complex I inhibition by mdivi-1.

## Materials and methods

### Reagents

We used mdivi-1 (Cat. No. 3982, 30 mM stock) and BAY-876 (Cat. No. 6199) from Tocris Bioscience. We used 6-ANA (sc-278446) and rotenone (Cat. No. 3616) from Santa Cruz Biotechnology (SCBT). We used uridine (Cat. No. U2275) from US Biological Life Sciences. We used galactose (Cat. No. G0750) from Sigma-Aldrich, fructose (Cat. No. F0127) and crystal violet (Cat. No. C6158) from Sigma Life Sciences, antimycin-A (Cat. No. A8674) and fibronectin (Cat. No. F0895) from Sigma-Aldrich. We used pyruvate (Cat. No. 11360-070) and glutamine (Cat. No. 25030-081) from Gibco Life Technologies. Stock solutions of all compounds were prepared in dimethyl sulfoxide (DMSO).

### Cell culture

Breast cancer cell lines (MCF-7 and MDA-MB-231) were purchased from the American Tissue Culture Collection (ATCC). Colon cancer cell lines HCT116 wild-type (WT) and HCT116 DRP1-KO (knockout) were kindly provided by Dr. Karbowski from the University of Maryland. These cells were grown in Dulbecco’s modified Eagle’s medium (DMEM), containing 25 mM glucose (HG) and 4 mM glutamine supplemented with 10% fetal bovine serum (FBS) and antibiotic/antimycotic-1X. In addition, the MDA-MB-231 oxidative subpopulations and MDA-MB-231-Rho-0 cells (Rho-0) were generated as described^20^. MDA-MB-231 oxidative subpopulations were grown in DMEM without glucose containing 10 mM galactose and 4 mM glutamine. Rho-0 cells were grown in DMEM-HG containing 1 mM pyruvate, 50 µg/mL uridine, and supplemented with 10% FBS. Osteosarcoma cell lines 143Bwt and 143BΔcytb were kindly provided by Dr. N. S. Chandel from Northwestern University. These cells were cultured in DMEM-HG containing 1 mM pyruvate, 100 μg/mL uridine, and supplemented with 10% FBS. All cancer cells were maintained in a humidified atmosphere at 37 °C and 5% CO_2_.

### Analysis of the number of floating and total viable cancer cells

The fraction of viable floating cancer cells relative to the total viable cell population was determined as follows: % viable floating cells = 100 × (number viable floating cells)/(number viable floating cells + number viable adherent cells) as described^21^.

### SDS-PAGE and western blotting analysis

Cancer cells were lysed with RIPA buffer (Millipore #20-188) supplemented with protease and phosphatase inhibitors (Sigma-Aldrich #P2714, Roche PhosSTOP™). Proteins were separated in 10% SDS–polyacrylamide gels and transferred to polyvinylidene difluoride membranes (Millipore). Blocking was performed at room temperature for 1 h in 5% BSA, and membranes were incubated overnight at 4 °C with the primary antibodies: rabbit monoclonal anti-DRP1 (Cat. No. 8570) from CST (Cell Signaling Technology, USA) and anti-β-actin (sc-47778) from SCBT. Then, the membranes were incubated for 1 h at room temperature with the secondary antibodies conjugated to horseradish peroxidase. Chemiluminescence detection used ECL Plus reagent (Pierce), and a series of timed exposure images were acquired with a FluorChem Q system (ProteinSimple) to ensure that densitometric analyses by ImageJ software (NIH, USA) were performed at exposures within the linear range.

### Transwell migration assay

Migration assay was performed in Boyden Chambers of 6.5 mm diameter and 8-μm pore size, according to the manufacturer’s instructions (Transwell™ Costar). Briefly, the bottom of each insert was coated with 0.1 mL of 2 μg/mL fibronectin for 16 h at 4 °C. Cells (1.0 × 10^4^) were suspended in an FBS-free cell culture medium and seeded onto the top of each chamber insert. DMEM-HG supplemented with 10% FBS was added to the bottom chamber. After 2 h (MDA-MB-231 cells) and 16 h (MCF-7 cells), inserts were removed and washed, and cells adhered to the bottom side of the inserts were stained with 0.5% crystal violet. The number of migrated cells was determined under an inverted microscope and photographed.

### Cell cycle analysis

To estimate cell cycle distribution, cellular DNA contents were measured by flow cytometry as described in^22^. Cancer cells were incubated with DMSO (Control) or 50 µM mdivi-1 for 24 h. All samples were analyzed for cell cycle distribution using a BD FACS Canto II flow cytometer and the BD FACSDiva™ software (San Jose, CA, USA). For data analysis, the FlowJo software v8.8 was used. Data were reported as the percentage of cells in each cell cycle phase, gating the cell populations with propidium iodide (PI) fluorescence.

### DNA isolation and quantification of mitochondrial DNA (mtDNA)

The mitochondrial DNA (mtDNA) copy number was quantified as follows: after DNA isolation, real-time quantitative PCR was performed in triplicates on 96-well reaction plates (Bio-Rad) in final volumes of 25 μL. Each reaction contained 15 ng of DNA template, 1X Power SYBRGreen PCR Master Mix (Applied Biosystems) and 0.5 μM of forward and reverse primers. The mtDNA and nuclear DNA were amplified using primers specific to regions of human t-RNA and B2 microglobulin genes respectively. t-RNA forward sequence (5′-CACCCAAGAACAGGGTTTGT-3′) and reverse sequence (5′-TGGCCATGGGTATGTTGTTA-3′). B2 microglobulin forward sequence (5′-TGCTGTCTCCATGTTTGATGTATCT-3′) and reverse sequence (5′-TCTCTGCTCCCCACCTCTAAGT-3′). Changes in the mtDNA copy number were determined by using the 2^−ΔΔCt^ method^23^.

### Viability assay

Cancer cells were stained with 50 µL 0.5% crystal violet for 1 h. Then, 200 µL methanol was added to each well, and the absorbance was measured at 570 nm^24^. For experiments using a flow cytometer, the cell viability/death was assayed using 1 μM SYTOX™ Blue Nucleic Acid Stain (Invitrogen™, #S11348).

### Seahorse XFe96 analyzer

Multiparameter metabolic analysis of breast cancer cells was performed in an extracellular flux analyzer XFe96 (Agilent, USA). MDA-MB-231 and MCF-7 cells were seeded on XFe96 multi-well plates and kept overnight at 37 °C in 5% CO_2_ with DMEM-HG. The next day, the cell culture medium was replaced with Agilent seahorse XF DMEM-1X medium (pH 7.4) (with 5 mM HEPES) with 10 mM glucose 1 h before the assay. Mitochondrial function was evaluated using 1 µM oligomycin (Oligo), 250 nM FCCP, 1 µM rotenone (Rot), and 1 µM antimycin-A (AA).

### Determination of ROS levels

The generation of intracellular oxidative stress was determined using the dihydroethidium (DHE) probe (Invitrogen™, D1168). Cancer cells were grown in complete cell culture medium, seeded in 6-well plates, and allowed overnight to attach. Then, cells were exposed for 1 or 4 h to DMSO or 50 µM mdivi-1, at the end of the exposure time, cell culture medium was replaced by a solution containing 5 µM DHE in PBS-1X and incubated for 30 min in the dark. After this, cells were washed, trypsinized, resuspended in 200 µL of PBS-1X, and measured by CytoFLEX™ V3-B3-R3 flow cytometer (Beckman Coulter).

### Measurement of mitochondrial parameters (∆Ψm)

Mitochondrial membrane potential in intact cells was determined using the potentiometric probe tetramethyl rhodamine methyl ester (TMRE) (Invitrogen, cat# T669). MDA-MB-231 cells (1.2 × 10^5^) were treated with DMSO (Control) or 50 µM mdivi-1 for 24 h. Cancer cells were washed with PBS-1X and incubated with 5 nM TMRM for 30 min. Cells were collected, washed, re-suspended, and acquired on a CytoFLEX™ V3-B3-R3 flow cytometer (Beckman Coulter). The data was analyzed in the FlowJo vX.0.7 software (Tree Star, Inc.).

### Glucose uptake

Glucose uptake was assessed using the fluorescent substrate 2-(N-(7-nitrobenz-2-oxa-1,3-diazol-4-yl)amino)-2-deoxyglucose (2-NBDG)^25^. Cancer cells were treated with DMSO (Control) or 50 µM mdivi-1 for 24 h. Then, 50 µM 2-NBDG was added, and cells were incubated during 30 min at 37 °C in the dark. After this time, cell was washed, collected, and resuspended in 200 µL of cold PBS-1X. The 2-NBDG fluorescence was quantified using a CytoFLEX™ V3-B3-R3 flow cytometer (Beckman Coulter). The data was analyzed with the FlowJo vX.0.7 software (Tree Star, Inc.).

### Invasiveness assay

We generated MDA-MB-231 spheroids as described^26^. Briefly, a 96-well plate was treated with 1% w/v low melting point agarose (Cleaver Scientific, UK) solution in MEM (Gibco Life Technologies, USA) without FBS to generate a low adhesion surface. After solidification of the agarose, 1.0 × 10^4^ cells plus 10 μg/mL collagen I (Gibco Life Technologies, USA) were added per well. The plate was centrifuged at 1000 rpm for 10 min and maintained under standard cell culture conditions. After 5–7 days, when spheroids looked compact, well-defined, and with a regular shape, they were transferred to a 12-well invasion plate. The invasion plate was pre-treated with 1.25 mL of 2.2 mg/mL collagen I for 5 min, followed by washing and placed in a collagen I gel matrix made of 2 mL of 3 mg/mL collagen I supplemented with 250 μL of MEM-10X (Gibco Life Technologies, USA) and 600 μL 0.1 M NaOH (Merck Millipore, USA). The whole procedure was performed at ice-cold temperatures, including the materials.

### Clonogenic formation assay

Adherent and floating cells (1 × 10^3^) were seeded into 6-well plates, and their clonogenic potential was evaluated 7–8 days after re-seeding. Throughout this period, the cell culture medium was replaced every 3 days. The clonogenic potential of the re-seeded cells was quantified using crystal violet staining. The cell culture medium was discarded, and the colonies were washed with PBS-1X and treated with 0.5% crystal violet for 45 min. Following incubation, the culture plates were washed with H_2_O and allowed to dry at room temperature, and images of the colonies were captured using a transilluminator. Finally, the colonies were counted using ImageJ software (NIH, USA).

### MitoTracker™ staining

HCT116 cells were incubated with 50 µM mdivi-1 for 4 h. During the final 20 min of incubation, the cells were treated with 100 nM MitoTracker™ Deep Red FM Dye. Then, HCT116 cells were washed once with PBS-1X and fixed using PFA 4% (in PBS-1X) for 10 min. Images were acquired using a Leica TCS SP8 Confocal Laser Scanning Microscope (Leica™) with a 63 × objective. The obtained images were analyzed and quantified using ImageJ (NIH) with the mitochondria-analyzer plugin (available at https://github.com/AhsenChaudhry/Mitochondria-Analyzer). Finally, Hoechst was used to label nuclear DNA of cells.

### Statistical analysis

All statistical analyses were performed using GraphPad Prism 8 version 8.0.2 (GraphPad Software, San Diego, CA, USA). The data are presented as mean ± SEM of three or more independent experiments. Statistical analysis was performed using unpaired *t* tests, one-way analysis of variance (ANOVA) with Tukey´s multiple comparisons test. Significance level of statistical tests: *p < 0.05; **p < 0.01; ***p < 0.001; ***p < 0.0001 and ns = not significant.

### Ethics approval

Universidad Mayor’s Bioethics and Biosafety Committee has approved work with human cancer cell lines.

## Results

### Mdivi-1 induces detachment of viable MCF-7 and MDA-MB-231 breast cancer cells

**Figure 1:**
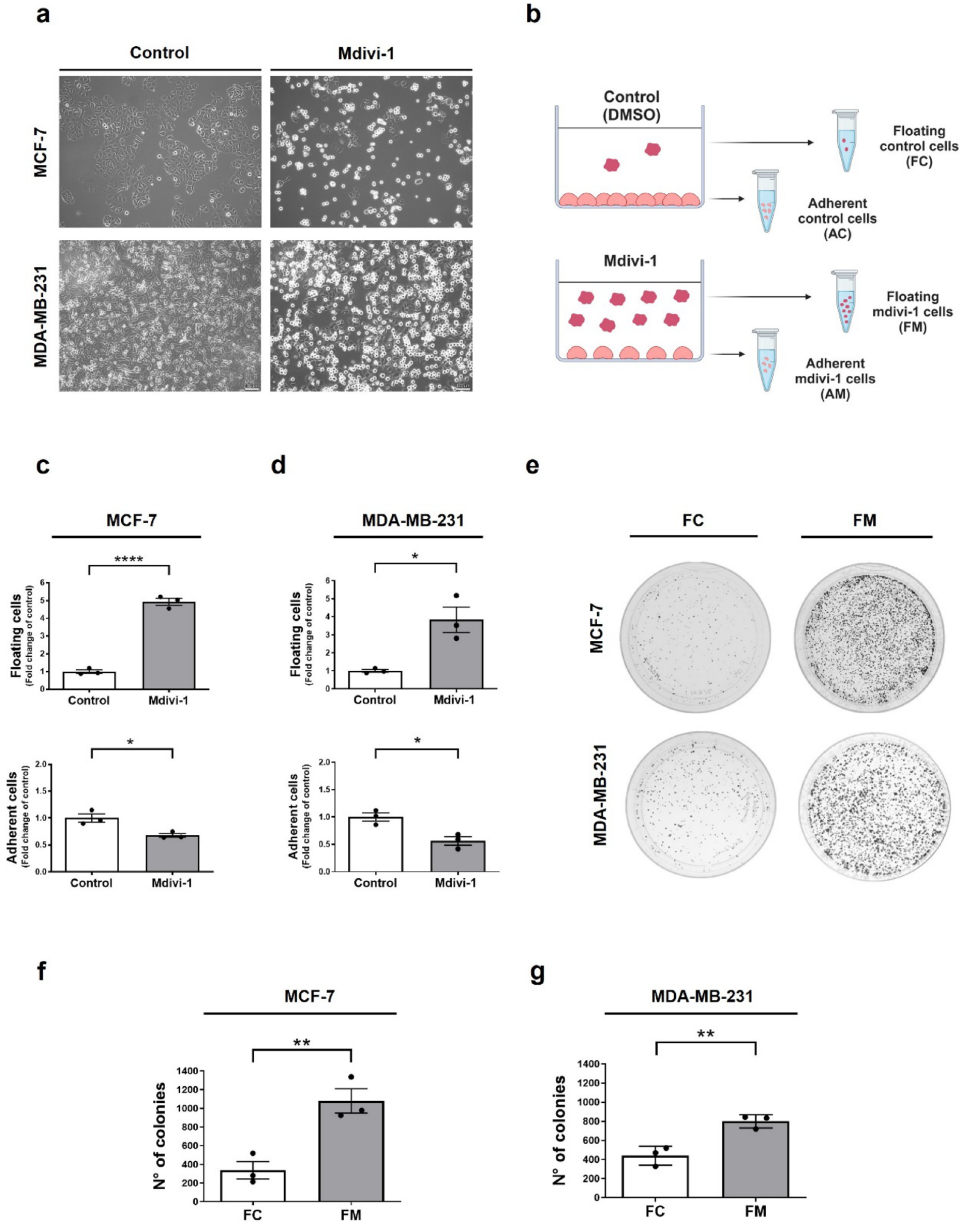
Mdivi-1 induces detachment of viable breast cancer cells. (**a**) MCF-7 and MDA-MB-231 cells were photographed, and the cellular morphology was documented using a phase-contrast microscope. (**b**) Schematic representation of the separation of floating and adherent cells into four cell populations: FC, FM, AC, and AM (Created with BioRender.com). (**c**) MCF-7 and (**d**) MDA-MB-231 cells were treated with 50 μM mdivi-1 for 24 h. Then, the fraction of viable floating cells relative to the total viable cell population and viable adherent cells was determined. (**e**), (**f**) MCF-7 and (**e**), (**g**) MDA-MB-231 cells were treated with 50 μM mdivi-1 for 24 h. Next, a clonogenic assay was performed using only the floating cells, FC and FM, isolated from the cell culture medium. FC and FM cells were re-seeding and resuspended in mdivi-1-free cell culture medium. After seven days of growth, the number of colonies were measured using ImageJ software. The scale bar corresponds to 100-μm. Results represent means ± SEM. *p < 0.05; **p < 0.01; ****p < 0.0001.

Mdivi-1, a mitochondrial division inhibitor, is well-known for its anti-proliferative effects on various tumor cells in vitro and in vivo^16,17,27^. Due to its promising therapeutic potential in inhibiting tumor growth, mdivi-1 is considered an attractive candidate for treating solid tumors^28^. Notably, we have observed that treatment with 50 μM mdivi-1 for 24 h induces detachment of adherent MCF-7 and MDA-MB-231 cells from the bottom of standard cell culture plates, as shown in Fig. 1a. Similar results are observed using 25 μM mdivi-1 for 48 h (Fig. S1a). Both concentrations of mdivi-1 used in this study have been used in vitro previously^17,19^. MCF-7 and MDA-MB-231 cells become round-shaped before cell detachment, which begins to be observed 6 h after mdivi-1 treatment (Fig. S1b). Whether these detached “floating” cells are viable or dying is unknown.

To assess the viability of the floating cells, we used the trypan blue assay to determine the fraction of viable cells relative to the total viable cell population as described^21^. As shown in Fig. 1b, floating and adherent cells were separately collected and counted, resulting in four distinct cell populations: floating control cells (FC), floating cells induced by mdivi-1 (FM), adherent control cells (AC), and adherent cells that remained after treatment with mdivi-1 (AM). Interestingly, mdivi-1 increased the detachment of viable MCF-7 (Fig. 1c) and MDA-MB-231 (Fig. 1d) cells but also decreased the number of viable adherent cells to about 50% compared to the control (Fig. 1c,d). These findings are consistent with previous studies associating mdivi-1 with decreased viability in cancer cells^17,27,28^.

To confirm the viability of floating MCF-7 and MDA-MB-231 cells induced by mdivi-1, we conducted a clonogenic assay using the FC and FM populations (Fig. 1e). After seven days of growth, the number of FM colonies was greater than FC colonies likely due to the substantial number of cells in suspension in the FM population before seeding (Fig. 1f,g). Both FC and FM from MCF-7 and MDA-MB-231 cells were able to re-attach, and cell detachment induced by mdivi-1 induce once again (Fig. S1c,d).

To exclude the possibility that cell detachment is due to an alteration in the surface of the polystyrene chains of cell culture plates by mdivi-1, we coated the plates with extracellular matrix (ECM) protein fibronectin before seeding the cells. As observed in Fig. S1e,f, mdivi-1 also induces cell detachment of viable MCF-7 and MDA-MB-231 cells seeded in fibronectin-coated plates. Additionally, we determine that both FC and FM from MDA-MB-231 cells exhibited elevated levels of intracellular ROS (i.e., superoxide and hydrogen peroxide) (Fig. S1g), as well as increased mitochondrial membrane potential (∆Ψm) compared to AC and AM (Fig. S1g), which could reflect metabolic and redox alterations to survive after cell detachment^29^. Altogether, these results show that mdivi-1 induces a bulk of floating viable breast cancer cells that maintain the capacity to re-attach upon re-seeding.

### The floating MCF-7 and MDA-MB-231 cells induced by mdivi-1 maintain the proliferative and clonogenic potential upon re-seeding

**Figure 2:**
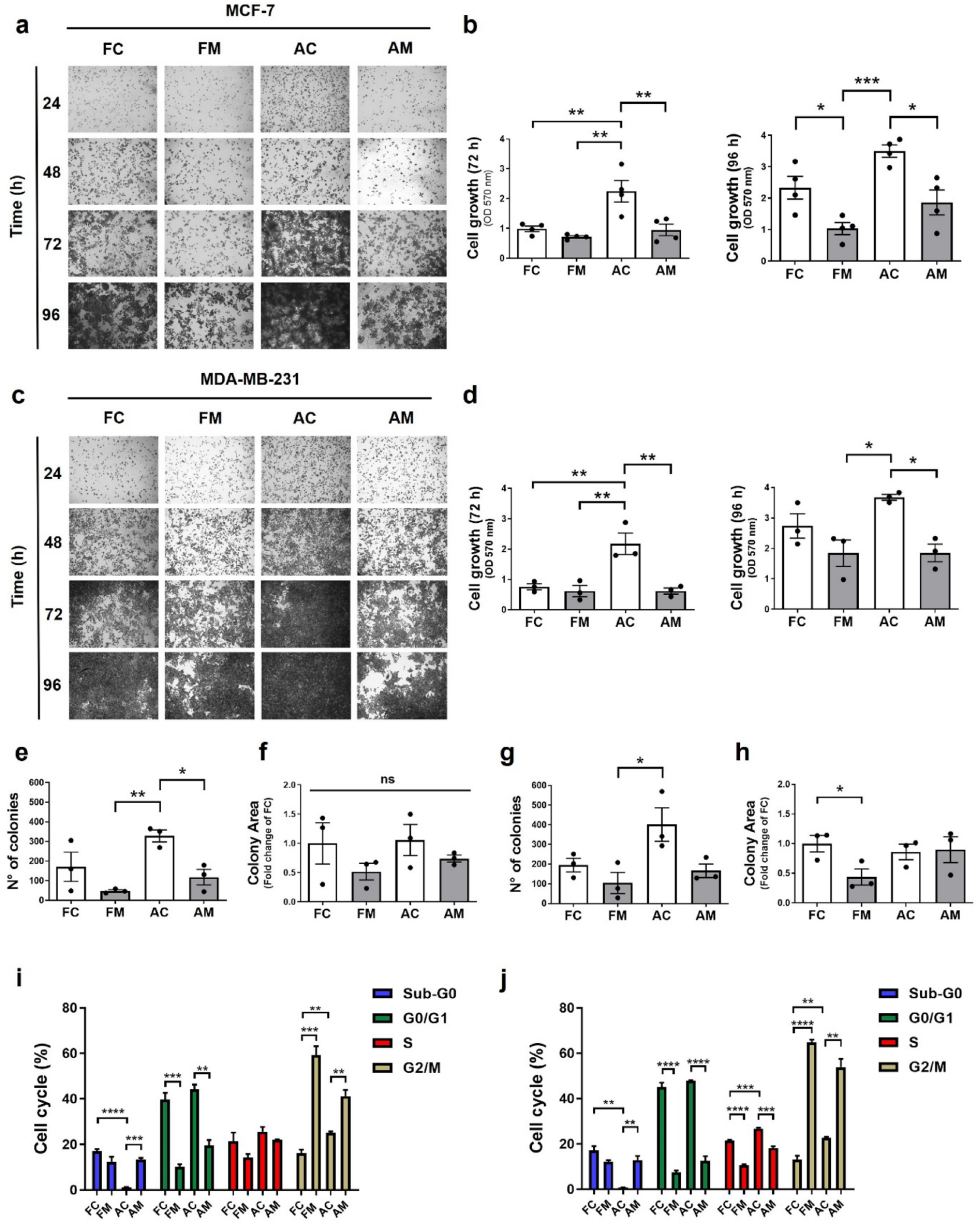
The floating breast cancer cells induced by mdivi-1 maintain the proliferative and clonogenic potential upon re-seeding. (**a**), (**b**) MCF-7 and (**c**), (**d**) MDA-MB-231 cells were treated with 50 μM mdivi-1 for 24 h. Floating and adherent cancer cells were collected and re-seeded in mdivi-1-free cell culture medium for 24, 48, 72, and 96 h. Then, a proliferation assay was performed using crystal violet. (**e**), (**f**) MCF-7 and (**g**), (**h**) MDA-MB-231 cells were treated with 50 μM mdivi-1 for 24 h. Next, four cell populations were collected and re-seeding to carry out the clonogenic assay. After seven days of growth, the number and area of colonies were measured using ImageJ software. (**i**) MCF-7 and (**j**) MDA-MB-231 cells were treated with 50 μM mdivi-1 for 24 h. Floating and adherent cells were collected, and then a cell cycle assay analysis was performed. Results represent means ± SEM. *p < 0.05; **p < 0.01; ***p < 0.001.

Since FM from MCF-7 and MDA-MB-231 cells are viable in a suspension state and can re-attach upon re-seeding, we would like to know if they maintain their proliferative and clonogenic potential. To evaluate this, floating and adherent MCF-7 (Fig. 2a,b) and MDA-MB-231 (Fig. 2c,d) cells obtained after treatment with 50 μM mdivi-1 for 24 h were re-seeding and a proliferation analysis by crystal violet staining was performed at different times (Fig. S2a,b). At 72 h, FM MCF-7 (Fig. 2b) and MDA-MB-231 (Fig. 2d) cells showed a lower proliferation than AC cells. Furthermore, FC and AM cells present a lower proliferation than AC cells. For example, at 96 h, FM MCF-7 cells (Fig. 2b) showed a lower proliferation than FC and AC cells. Also, AM cells present a diminished proliferation concerning AC cells (Fig. 2b). In MDA-MB-231 cells, FM and AM cells present a lower proliferation than AC cells. However, FC and AC cells exhibited similar proliferation, like in MCF-7 cells (Fig. 2d). Importantly, it should be noted that all floating cells exhibited growth potential.

On the other hand, we performed a clonogenic assay to assess the clonogenic potential of floating and adherent cells obtained after treatment with mdivi-1 (Fig. S2c,d). After seven days, FM and AM from MCF-7 (Fig. 2e) and FM from MDA-MB-231 (Fig. 2g) cells present fewer colonies than AC. When we measured the area of the colonies, no differences between the four MCF-7 cell populations were found (Fig. 2f). However, in MDA-MB-231 cells (Fig. 2h), we found that FM colonies present a smaller area than FC. These observations suggest that even though mdivi-1 decreases FM and AM proliferative and clonogenic capacity, these cell populations retain their proliferative and clonogenic potential in vitro after re-seeding.

To determine whether this decrease in cell proliferation is due to alterations in the cell cycle, we performed a cell cycle analysis by flow cytometry to determine at which phase of the cell cycle are the four cell populations. Thus, we found that FM and AM from MCF-7 and MDA-MB-231 cells present a more significant percentage of cells in the G2/M phase than FC and AC (Fig. 2i,j), unlike FC and AC from MCF-7 and MDA-MB-231 cells present a more significant percentage of cells in the G0/G1 phase than FM and AM cells (Fig. 2i,j). These results show that mdivi-1 prevented completion of the mitotic program, possibly by G2 arrest in breast cancer cells. The increase in Sub-G0 phase observed in the floating fractions (FC and FM) suggested that some cells undergo apoptosis in both MCF-7 and MDA-MB-231 cells. Thus, we determine cell death in the floating fraction with SYTOX™ Blue dead cell stain and flow cytometry. As shown is Fig. S2e, no difference in the percentage of cell death between FC and FM where find.

### The floating MCF-7 and MDA-MB-231 cells induced by mdivi-1 maintain the migratory and invasive potential upon re-seeding

**Figure 3:**
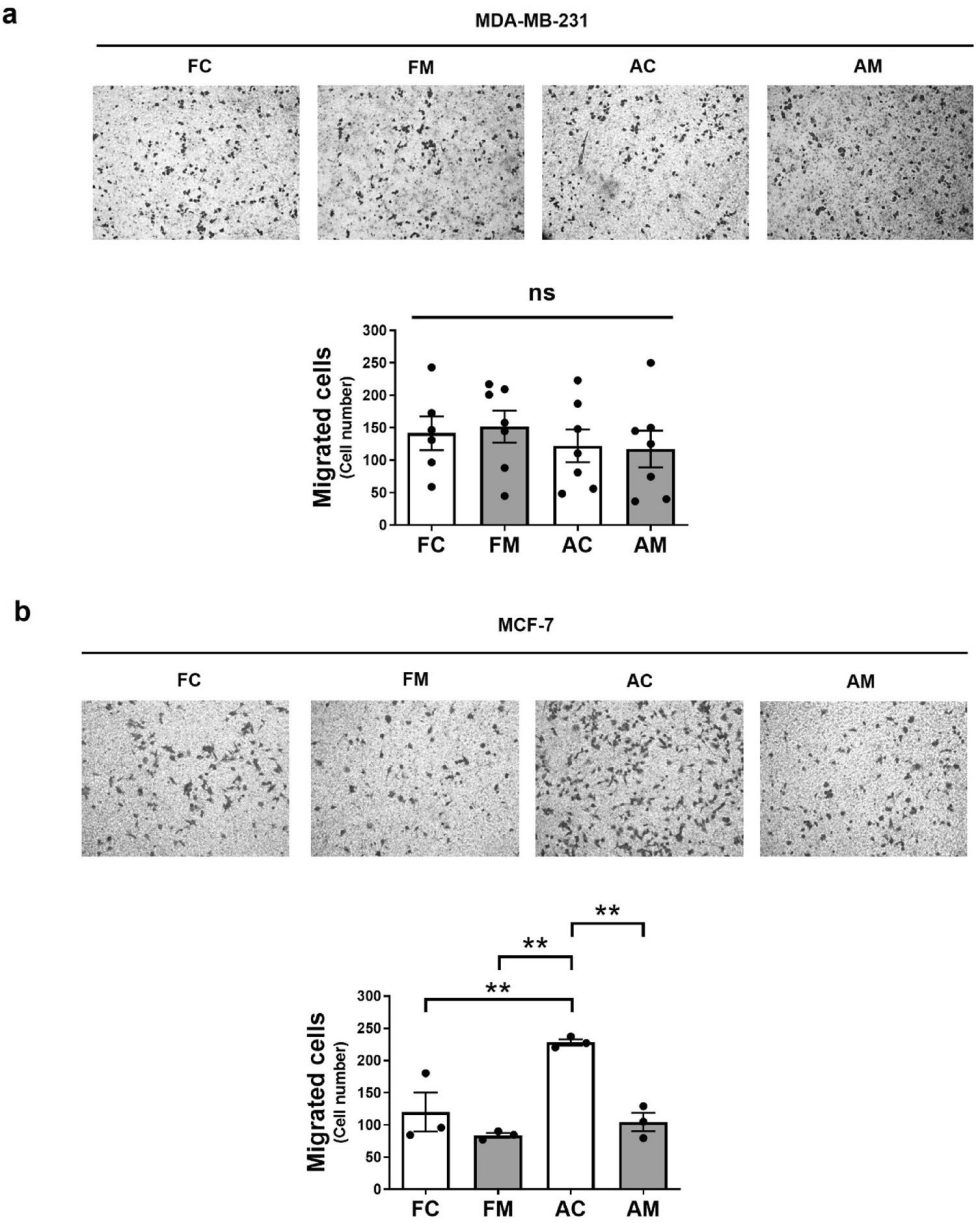
The floating breast cancer cells induced by mdivi-1 maintain the migratory potential upon re-seeding. (**a**) MDA-MB-231 and (**b**) MCF-7 cells were treated with 50 μM mdivi-1 for 24 h. Floating and adherent cells were collected, and a transwell migration assay was performed. The four cell populations were photographed, and in vitro transmigration was documented using a phase-contrast microscope. Results represent means ± SEM. **p < 0.01; ns = not significant.

Since mdivi-1 induces detachment of viable breast cancer cells capable of proliferating after re-seeding, we wonder if they also maintain their migratory and invasive potential. Therefore, we measured in vitro transmigration using the transwell assay after re-seeding the four MCF-7 and MDA-MB-231 cell populations obtained after treatment with 50 μM mdivi-1 for 24 h. In MDA-MB-231 cells, no differences are observed between FC, FM, AC, and AM populations (Fig. 3a). In MCF-7 cells, FC, FM, and AM present a lower migration than AC cells (Fig. 3b). However, FC and FM cells presented a similar migratory potential. Additionally, we conducted an in vitro invasiveness assay using MDA-MB-231 cell spheroids to assess the invasive capabilities of the four cell populations. After five days, no differences were observed (Fig. S3). These observations suggest that floating cells induced by mdivi-1 still retain their migratory and invasive potential in vitro after re-seeding.

### The detachment of viable MCF-7 and MDA-MB-231 cells induced by mdivi-1 is independent of DRP1

**Figure 4:**
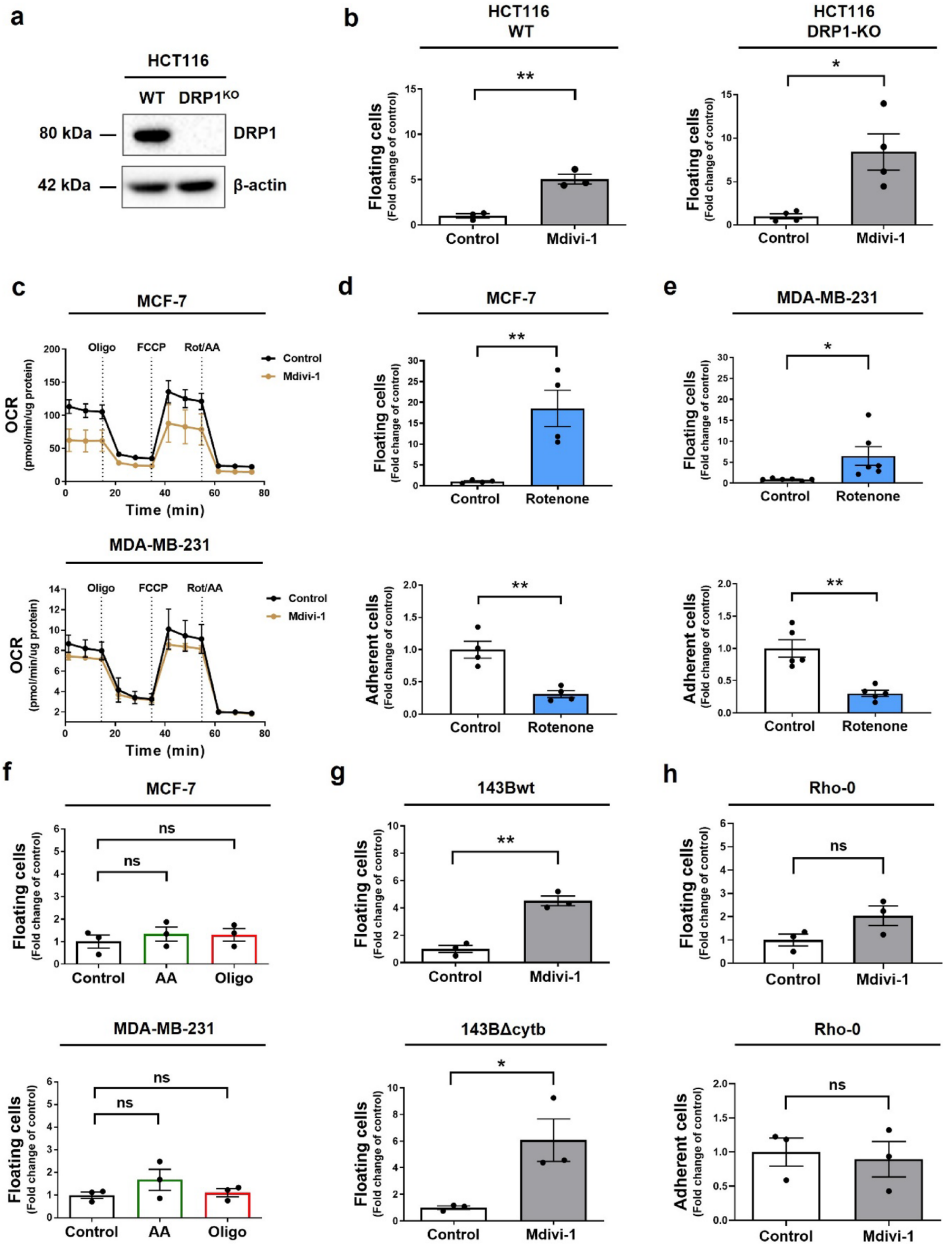
Mdivi-1 induces detachment of breast cancer cells by a DRP1-independent mechanism. (**a**) DRP1 and β-actin protein levels in WT and DRP1-KO HCT116 cells were determined by western blot (**b**) these cells were treated with 50 μM mdivi-1 for 24 h. Next, the fraction of viable floating cells relative to the total viable cell population was determined. (**c**) MCF-7 and MDA-MB-231 cells were treated with 50 μM mdivi-1 for 4 h. Then, a Mito Stress test was performed using a seahorse XFe96 analyzer. (**d**) MCF-7 and (**e**) MDA-MB-231 cells were treated with 10 μM rotenone for 24 h, and then, the fraction of viable floating and adherent cells was determined. (**f**) MCF-7 and MDA-MB-231 cells were treated with 5 μM antimycin-A (AA) and 5 μM oligomycin (Oligo) for 24 h. The fraction of viable floating cells was determined. (**g**) 143Bwt, 143BΔcytb and (**h**) Rho-0 cells were treated with 50 μM mdivi-1 for 24 h. Then, the fraction of viable floating and adherent cells was determined. Results represent means ± SEM. *p < 0.05; **p < 0.01; ns = not significant.

Mdivi-1 is a widely accepted inhibitor of DRP1^30^. To investigate whether the cell detachment induced by mdivi-1 is mediated through DRP1 inhibition, we treated with 50 μM mdivi-1 HCT116 colon cancer cells DRP1 knockout (DRP1-KO) and evaluated cell detachment (Figs. 4a and S4a). Unexpectedly, mdivi-1 led to the detachment of viable wild-type (WT) and DRP1-KO HCT116 cells (Fig. 4b) reducing the number of viable adherent cells to about 50% compared to the control (Fig. S4b). These findings strongly suggest that the detachment of viable cancer cells is not dependent on DRP1 inhibition. Noteworthy, a 4 h mdivi-1 (50 µM) treatment in both WT and DRP1-KO HCT116 cells did not affect the mitochondrial network (Fig. S4c).

Importantly, Bordt et al. reported that mdivi-1 inhibits mitochondrial complex I. Like rotenone, mdivi-1 inhibits NADH-supported oxygen consumption rate (OCR)^19^. Therefore, we assessed the effect of mdivi-1 on the mitochondrial respiration of breast cancer cells before cell detachment. Both MCF-7 and MDA-MB-231 cells treated for 4 h with 50 μM mdivi-1 showed a decreasing OCR trend (Fig. 4c). Analysis of basal, maximum, and ATP-link respiration are display in Fig. S4d. These observations suggest that a decrease in mitochondrial function by mdivi-1 may be the required signal that triggers the cell detachment of viable cells. We used rotenone, a classical complex I inhibitor, to evaluate whether cell detachment depends on complex I inhibition^19^. Surprisingly, like mdivi-1, rotenone induces the detachment of viable MCF-7 (Figs. 4d and S4e) and MDA-MB-231 (Figs. 4e and S4e) cells and decreases the number of viable adherent cells (Fig. 4d,e). Noteworthy, the cell detachment induced by rotenone was more potent than that induced by mdivi-1. This was not related to toxicity since no increase in cell death was observed (Fig. S4f).

To determine whether the cell detachment is specifically caused by the inhibition of complex I or is a general effect resulting from OXPHOS suppression, we conducted experiments using antimycin-A (AA) and oligomycin (Oligo) to inhibit complex III and V, respectively. However, as shown in Fig. 4f, these inhibitors did not induce the detachment of viable MCF-7 and MDA-MB-231 cells, suggesting that functional complex I inhibition mediates cell detachment. This observation is supported by the fact that both mdivi-1 (Fig. 4g) and rotenone (Fig. S4g,h) induce the detachment of viable 143BΔcytb cells, which have an active complex I but lack electron flow beyond complex III, resulting in an inability to perform OXPHOS^31^.

Finally, to provide evidence that the inhibition of active complex I mediates the detachment of viable cells, we generated mitochondrial DNA (mtDNA) depleted MDA-MB-231 (Rho-0) cells (Fig. S4i). As observed in Fig. 4h, treatment with mdivi-1 failed to induce either the detachment or the decreased of viable adherent Rho-0 cells. Altogether, these data suggest that the effect of mdivi-1 on cell detachment is independent of DRP1 and OXPHOS but dependent on the inhibition of active complex I.

### Mdivi-1 induces detachment of MCF-7 and MDA-MB-231 breast cancer cells in a glucose-dependent manner

**Figure 5:**
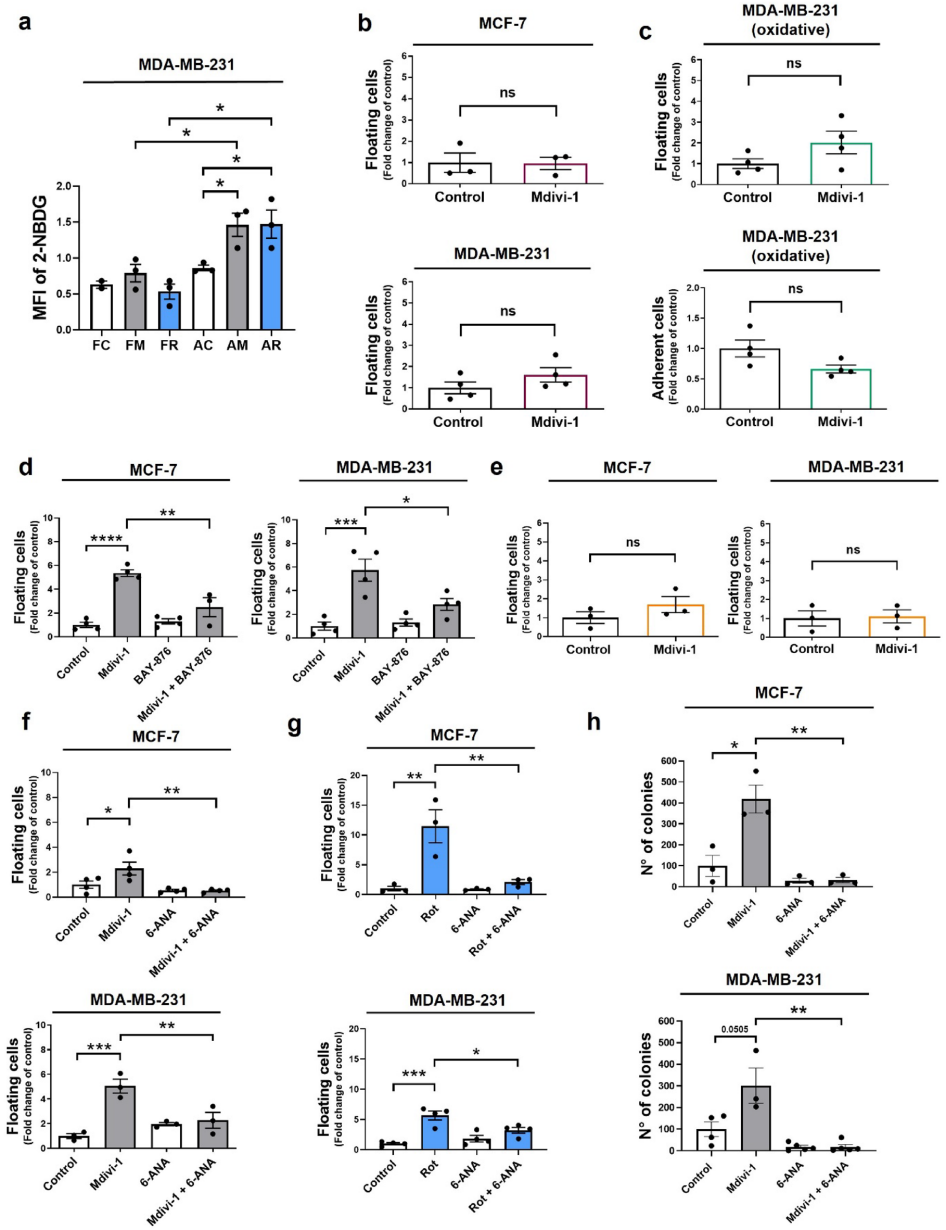
Mdivi-1 induces cell detachment in a glucose-dependent manner. (**a**) MDA-MB-231 cells were treated with 50 μM mdivi-1 and 10 μM rotenone for 24 h. Floating and adherent cells were collected, and a glucose uptake was assessed using the fluorescent substrate 2-NBDG. (**b**) MCF-7 and MDA-MB-231 cells were treated with 50 μM mdivi-1 for 24 h in a glucose-free cell culture medium. The fraction of viable floating cells was determined. (**c**) The oxidative subpopulation of MDA-MB-231 cells were treated with 50 μM mdivi-1 for 24 h. Next, the fraction of viable floating and adherent cells was determined. (**d**) MCF-7 and MDA-MB-231 cells were treated with 50 μM mdivi-1 plus 5 μM BAY-876 for 24 h. Then, the fraction of viable floating cells was determined. (**e**) MCF-7 and MDA-MB-231 cells were treated with mdivi-1 for 24 h in a glucose-free cell culture medium with 25 mM fructose. The fraction of viable floating cells was determined. (**f**) MCF-7 and MDA-MB-231 cells were treated with 50 μM mdivi-1 plus 50 μM 6-ANA for 24 h. The fraction of viable floating cells was determined. (**g**) MCF-7 and MDA-MB-231 cells were treated with 10 μM rotenone plus 50 μM 6-ANA for 24 h. The fraction of viable floating cells was determined. (**h**) MCF-7 and MDA-MB-231 cells were treated with 50 μM mdivi-1 plus 50 μM 6-ANA for 24 h. Then, a clonogenic assay was performed using only floating cells isolated from the cell culture medium. Next, cells were re-seeding and resuspended in mdivi-1-free cell culture medium. After seven days of growth, the number of colonies were measured using ImageJ software. Results represent means ± SEM. *p < 0.05; **p < 0.01; ***p < 0.001; ns = not significant.

To better understand the metabolic consequences of complex I inhibition by mdivi-1 and rotenone, we used a fluorescently labeled deoxy-glucose analog, 2-NBDG, to track glucose uptake in floating and adherent cells. In MDA-MB-231 cells, AM and AR increased 2-NBDG uptake compared to AC cells (Fig. 5a). However, neither FM nor rotenone-induced floating cells (FR) showed an increase in 2-NBDG uptake compared to FC cells (Fig. 5a). These results suggest increased glucose uptake may be a prerequisite for cell detachment induced by complex I inhibition in adherent cells.

Interestingly, the cell detachment induced by mdivi-1 in MCF-7 and MDA-MB-231 cells does not occur when there is no glucose in the cell culture medium (Fig. 5b). Moreover, mdivi-1 failed to induce cell detachment in a subpopulation of MDA-MB-231 (Fig. 5c) cells that had been cultured through several passages without glucose and had adapted to a highly oxidative metabolism^32^. Furthermore, using BAY-876, a small-GLUT1 glucose transport inhibitor, significantly reduced cell detachment in viable MCF-7 and MDA-MB-231 cells compared to treatment with mdivi-1 alone (Fig. 5d). BAY-876 did not induce cell death in MDA-MB-231 cells (Fig. S2e). This evidence indicates that mdivi-1-induced cell detachment in viable breast cancer cells depends on glucose in the cell culture medium. To determine whether this is a common feature induce by complex I inhibition, we used BAY-876 in rotenone treated MDA-MB-231. Interestingly, BAY-876 fail to prevent the cell detachment in this cell line (Fig. S5), which suggests that the glucose requirement is specific of mdivi-1 treated cells.

**Figure 6:**
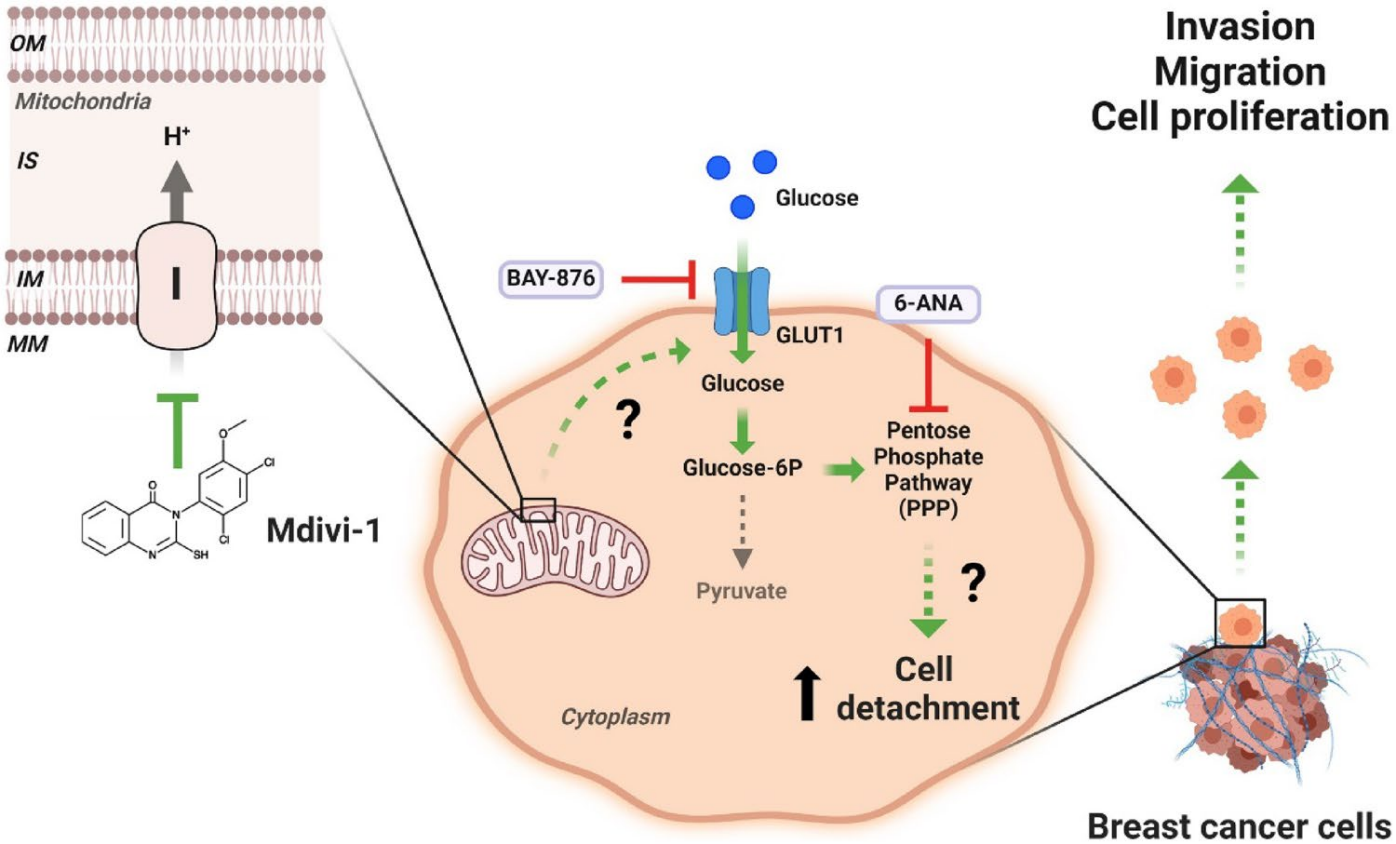
Model of detachment of viable human breast cancer cells induced by mdivi-1. The inhibition of mitochondrial complex I by mdivi-1 triggers the detachment of viable breast cancer cells. These cells maintain their proliferative, migratory, and invasive potential once re-seeding. The effect of mdivi-1 on the cell detachment is independent of DRP1 but dependent on the pentose phosphate pathway (PPP) upon complex I inhibition OM: Outer Mitochondrial Membrane, IS: Intermembrane Space, IM: Inner Mitochondrial Membrane, MM: Mitochondrial Matrix, I: Mitochondrial complex I. (Created with BioRender.com).

On the other hand, we wonder whether replacing fructose for glucose in the cell culture medium might also allow cell detachment induced by mdivi-1. Surprisingly, the cell detachment did not occur when we used a glucose-free medium supplemented with fructose (Fig. 5e). Fructose entry into the glycolytic pathway bypassed the limiting reactions of glycolysis (i.e., formation of glucose-6-phosphate and fructose-1,6-bisphosphate)^33^. Glucose-6-phosphate, the phosphorylated form of glucose, plays a crucial role in the pentose phosphate pathway (PPP), essential for cancer growth and metastasis^34^. We hypothesized that a glucose-fueled PPP might favor cell detachment induced by mdivi-1. Surprisingly, the detachment of MCF-7 (Fig. 5f) and MDA-MB-231 (Fig. 5g) cells induced by mdivi-1 or rotenone significantly decreased when we added 6-aminonicotinamide (6-ANA), a PPP inhibitor^35^. Additionally, a clonogenic assay using the floating cells from MCF-7 and MDA-MB-231 (Fig. 5h) cells treated with mdivi-1 plus 6-ANA revealed that after seven days, these floating cells had formed fewer colonies than FM colonies (Fig. 5h). Nevertheless, 6-ANA did not increase cell death (Fig. S2e). Overall, our data suggest that mdivi-1 treatment promotes in vitro detachment in viable breast cancer cells, facilitated by the PPP pathway in the context of complex I inhibition (Fig. 6).

## Discussion

Mitochondrial division inhibitor (mdivi-1) has been shown to decrease the viability of several tumor cells, including breast cancer cells^28^. However, this reduction could be associated with the induction of overlooked viable floating cancer cells^21^. Usually, the focus of most colorimetric (i.e., MTT, MTS, XTT, crystal violet), flow cytometry viability and proliferation assays focus on adherent cells, while floating cells are often neglected. Consequently, high-throughput assays might overlook drug effects related to the detachment of viable cancer cells. Our research presents new insights into the underexplored area of floating cancer cell viability, suggesting a link between cell proliferation inhibition and cell detachment induction.

Cancer cells have been observed to spontaneously detach and re-attach, suggesting a tightly regulated dynamic process^36^. Metastasis is a complex process known as the “metastatic cascade”^10^, involving multiple stages: cell invasion, migration, detachment, dissemination, and colonization in the target organ^10,37^. Notably, mdivi-1 appears to facilitate one critical stage of this process: the detachment of viable human breast cancer cells from the extracellular matrix (ECM). Several studies have highlighted the significance of circulating tumor cells (CTCs) in cancer metastasis. CTCs are tumor cells shed from the primary tumor and carried by the circulatory or lymphatic systems^11^. CTCs metastatic capacity correlates with a partial compromise mitochondrial function^38^. Thus, the reversible inhibition that mdivi-1 exert on complex I^19^ and the resultant decrease in mitochondrial function can explained the generation of the viable floating cells observed in this work. CTC as well as our breast cancer cell lines treated with mdivi-1, has a demonstrate ability to evade anoikis, a programmed cell death mechanism triggered by loss of cell attachment^39^. Additionally, in CTCs from breast cancer patients, upregulation of glucose-6-phosphate dehydrogenase (G6PD), a key enzyme in the pentose phosphate pathway (PPP), has been correlated with tumor metastasis and progression^40^. Given that our results indicate that mdivi-1-induced cell detachment depends on glucose and the PPP (Fig. 5d,f), it can be inferred that floating fraction induce by mdivi-1 may possess a high metastatic potential.

Mdivi-1 was initially reported to inhibit the GTPase activity of DRP1 in yeast^19^. However, Bordt et al. have questioned the efficacy of mdivi-1 as an inhibitor of DRP1, proposing that mdivi-1 is not especially effective in inhibiting the activity of recombinant human DRP1^19^. Additionally, several studies have underscored the DRP1-independent cellular effects induced by mdivi-1^17,19,41^. These findings have sparked a re-evaluation of existing literature and emphasized the need to identify potential targets for mdivi-1, enhancing our understanding of its applications. One such newly identified target is the mitochondrial complex I^19^.

Complex I, the largest respiratory complex of the electron transport chain (ETC), is crucial in oxidizing NADH and regulating oxidative phosphorylation (OXPHOS)^13^. Several studies have demonstrated changes in gene expression or mutations encoding complex I subunits in cancer, which provide an adaptive advantage to enhance cancer aggressiveness^13,42–44^. For instance, knockdown (KD) or mutations in core subunits like NDUFV1 or ND2 can promote metastatic behavior in cancer cells^43,45^. Along these lines, the KD of the accessory subunit NDUFB9 increases the migration and invasion of MDA-MB-231 cells^46^. However, results related to complex I assembly factors in cancer are contradictory. While low FOXRED1 expression correlates with poor prognosis in colorectal cancer, NUBPL KD decreases cell invasion^47^. Therefore, although complex I plays a significant role in cancer progression, the precise molecular mechanism continues to be elusive.

The anti-cancer activity of complex I inhibitors strongly supports the hypothesis that the whole blockage of complex I is detrimental to malignant progression^17,48,49^. However, this contrasts with findings in melanoma, where rotenone and metformin promote a pro-metastatic phenotype and tumor growth in vivo, respectively^15,50^. In line with this, our results support the idea that inhibition of complex I has a pro-metastatic effect. The dichotomy in the outcomes after complex I inhibition necessitates further understanding.

Mdivi-1 decreased mitochondrial oxidative metabolism in both lung and colorectal cancer cells, independent of DRP1 inhibition or induction of mitochondrial fusion^17^. In agreement, our results show the same trend without reaching significance. A recent report^51^ show that in HeLa cells and neurons, a 24 h treatment with 50 µM mdivi-1 did not significantly alter oxygen consumption rate (OCR). However, a shift in the metabolic profile was observed, indicating compromised oxidative energy metabolism and increased glycolysis. This treatment also increased mitochondrial mass, likely as a compensatory mechanism following complex I inhibition. Consequently, while the OCR appeared similar to control, normalizing the measurements by mitochondrial mass revealed a significant decrease in basal respiration, ATP synthesis-linked respiration, and maximal respiration. It is possible that a similar phenomenon is occurring in our system, which warrants further investigation in future studies. Additionally, mdivi-1 inhibits mitochondrial ATP production and increases glucose consumption in cancer cells. Notably, when combined with the glycolysis inhibitor 2-deoxy-D-glucose (2-DG), mdivi-1 exhibits lethality in MDA-MB-231 cells^52^. This decrease in mitochondrial ATP level and bioenergetics often triggers an upregulation of glycolysis as a compensatory mechanism to meet the energy demands of breast cancer cells. Indeed, it was previously shown that when complex I is inhibited with rotenone, breast cancer cells increase their glucose uptake and switch to a more glycolytic phenotype^53^. Interestingly, our experiments using the GLUT-1 inhibitor BAY-876 show inhibition of mdivi-1-induce floating fraction, but not effect on the one induce by rotenone. This discrepancy may have several explanations. One possibility is that rotenone’s effect, being more potent than that of mdivi-1, requires a higher concentration of BAY-876 for mitigation. Another explanation could be that rotenone’s mechanism involves glucose transporters other than GLUT1, such as GLUT4. Rotenone is known to induce the activation of AMP-activated protein kinase (AMPK)^54^. A critical consequence of AMPK activation is the stimulation of glucose uptake, facilitated by the increased translocation of GLUT4 to the plasma membrane^55^. More experiments are necessary to understand this difference.

In cancer cells, glycolysis activation may be accompanied by increased activity of the pentose phosphate pathway (PPP)^56^. The PPP plays a critical role in regulating cancer cell growth by supplying cells with ribose-5-phosphate and NADPH for the detoxification of intracellular reactive oxygen species (ROS), reductive biosynthesis, and ribose biogenesis^56^. Interestingly, paraquat, which decreases complex I activity^57^, up-regulates a key and rate limiting enzyme in the PPP such as the glucose-6-phosphate dehydrogenase (G6PD), increasing the metabolites within the PPP^58^. Our results using the PPP inhibitor 6-ANA point in the same direction, but further experiments need to be performed to establish in our system a direct connection between complex I inhibition and PPP.

On the other hand, the exclusive reliance on pharmacological tools in delineating the role of complex I activity as a mechanism associated with cell detachment induced by mdivi-1 and rotenone could be perceived as a limitation in our study. While the utilization of molecular interventions to explore the involvement of complex I may be a desirable augmentation, it is essential to acknowledge the intricate nature of such approaches. The KD of different subunits of complex I has been demonstrated to yield varying and occasionally contradictory outcomes in cancer cells. This underscores the complexity of the interplay between individual subunits and highlights the potential for nuanced, context-dependent responses. As such, caution must be exercised when interpreting results obtained through molecular interventions, as the diverse functions of complex I subunits may give rise to diverse cellular responses. In light of these considerations, our study, while primarily employing pharmacological tools, offers valuable insights into the initial understanding of the cell detachment induced by mdivi-1 and rotenone.

In summary, our findings establish that mdivi-1, a putative inhibitor of DRP1, induces the detachment of viable breast cancer cells while preserving their proliferative, clonogenic, migratory, and invasive potential. Notably, this effect operates through a DRP1-independent mechanism, intricately tied to glucose metabolism and reliance on the pentose phosphate pathway (PPP). Future research is necessary to precise how mdivi-1 disrupts complex I activity and to elucidate the specific role of the PPP in mediating the detachment of viable cells. Accordingly, our results provide valuable insights into potential therapeutic strategies for breast cancer intervention.

### Supplementary Information

Supplementary Figures.

## Data Availability

The data supporting this study’s findings are available from the corresponding authors (ESP and JCC) upon a reasonable request.
