# Supplementary material for: Mitochondrial division inhibitor (mdivi-1) induces extracellular matrix (ECM)-detachment of viable breast cancer cells by a DRP1-independent mechanism
Source: Sci Rep. 2024 Jun 19;14:14178. doi: 10.1038/s41598-024-64228-9 (PMC11187114; doi:10.1038/s41598-024-64228-9)
Supplement: Supplementary file 1 — Supplementary Figures. [file 41598_2024_64228_MOESM1_ESM.docx]

**Supplementary information**

**Mitochondrial division inhibitor (mdivi-1) induces extracellular matrix (ECM)-detachment of viable breast cancer cells by a DRP1-independent mechanism**

Eduardo Silva-Pavez^a,b†^, Elizabeth Mendoza^b,c^, Pablo Morgado-Cáceres^b,c^, Ulises Ahumada-Castro^b,c^, Galdo Bustos^b,c^, Matías Kangme-Encalada^b,c^, Amaia Lopez de Arbina^f^, Andrea Puebla-Huerta^b,c^, Felipe Muñoz^b,c^, Lucas Cereceda^d,e^, Manuel Varas-Godoy^g,h,i^, Yessia Hidalgo^d,e^ and J. Cesar Cardenas^b,c,j,k†^

^a^Facultad de Odontología y Ciencias de la Rehabilitación, Universidad San Sebastián, Bellavista, Santiago, Chile.

^b^Center for Integrative Biology, Faculty of Sciences, Universidad Mayor, Santiago, Chile.

^c^Geroscience Center for Brain Health and Metabolism, Santiago, Chile.

^d^IMPACT, Center of Interventional Medicine for Precision and Advanced Cellular Therapy, Santiago, Chile.

^e^Laboratory of Nano-Regenerative Medicine, Biomedical Research and Innovation Center (CIIB), Faculty of Medicine, Universidad de los Andes, Santiago, Chile.

^f^Biodonostia Health Research Institute, 20014, San Sebastián, Spain.

^g^Cancer Cell Biology Lab., Centro de Biología Celular y Biomedicina (CEBICEM), Facultad de Medicina y Ciencia, Universidad San Sebastián, Lota 2465, Santiago, Chile.

^h^Centro Ciencia & Vida, Fundación Ciencia & Vida, Avenida Del Valle Norte 725, Huechuraba, Santiago, Chile.

^i^Advanced Center for Chronic Diseases (ACCDiS), Faculty of Medicine, Universidad de Chile, Santos Dumont 964, Independencia, Santiago, Chile.

^j^Buck Institute for Research on Aging, Novato, USA.

^k^Department of Chemistry and Biochemistry, University of California, Santa Barbara, USA.

**^†^Correspondence:**

Dr. Eduardo Silva-Pavez

Facultad de Odontología y Ciencias de la Rehabilitación

Bellavista 7, Recoleta

Universidad San Sebastián

Santiago, Chile

email: [eduardo.silva@uss.cl](mailto:eduardo.silva@uss.cl)

Dr. J. Cesar Cardenas

Center for Integrative Biology

Camino la Pirámide 5750, Huechuraba

Universidad Mayor

Santiago, Chile

email: [julio.cardenas@umayor.cl](mailto:julio.cardenas@umayor.cl)


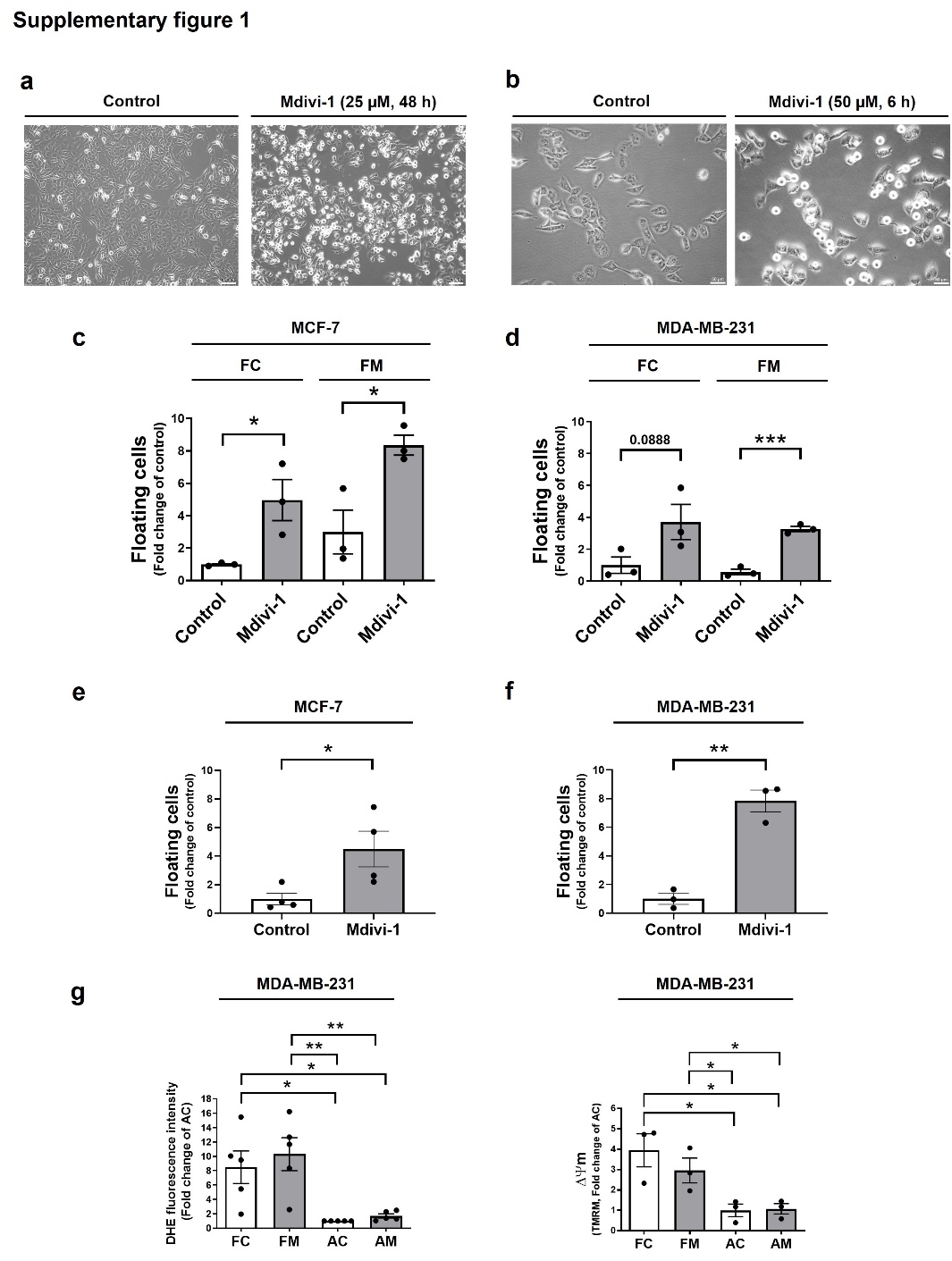


**Supplementary figure S1.** Mdivi-1 induces changes in cellular morphology and detachment without disrupting re-adhesion of floating breast cancer cells. (**a**) MDA-MB-231 cells were treated with 25 μM mdivi-1 for 48 h and photographed, and cellular morphology was documented using a phase-contrast microscope. (**b**) MDA-MB-231 cells were treated with 50 μM mdivi-1 for 6 h and photographed. (**c**) MCF-7 and (**d**) MDA-MB-231 cells were treated with 50 μM mdivi-1 for 24 h, and next, floating cells were re-seeding and resuspended in mdivi-1-free culture medium. 24 h after, cells were treated with 50 μM mdivi-1 for 24 h, and next, the fraction of viable floating cells was determined. (**e**) MCF-7 and (**f**) MDA-MB-231 were seeded in plates coated with 2 μg/mL fibronectin. After 24 h, these cells were treated with 50 μM mdivi-1 for 24 h. Then, the fraction of viable floating and adherent cells was determined. **(g)** MDA-MB-231 cells were treated with 50 μM mdivi-1 for 24 h. Floating and adherent cells were collected to determine DHE fluorescence intensity and mitochondrial membrane potential (∆Ψm). Results represent means ± SEM. *p< 0.05; **p< 0.01; ***p< 0.001.


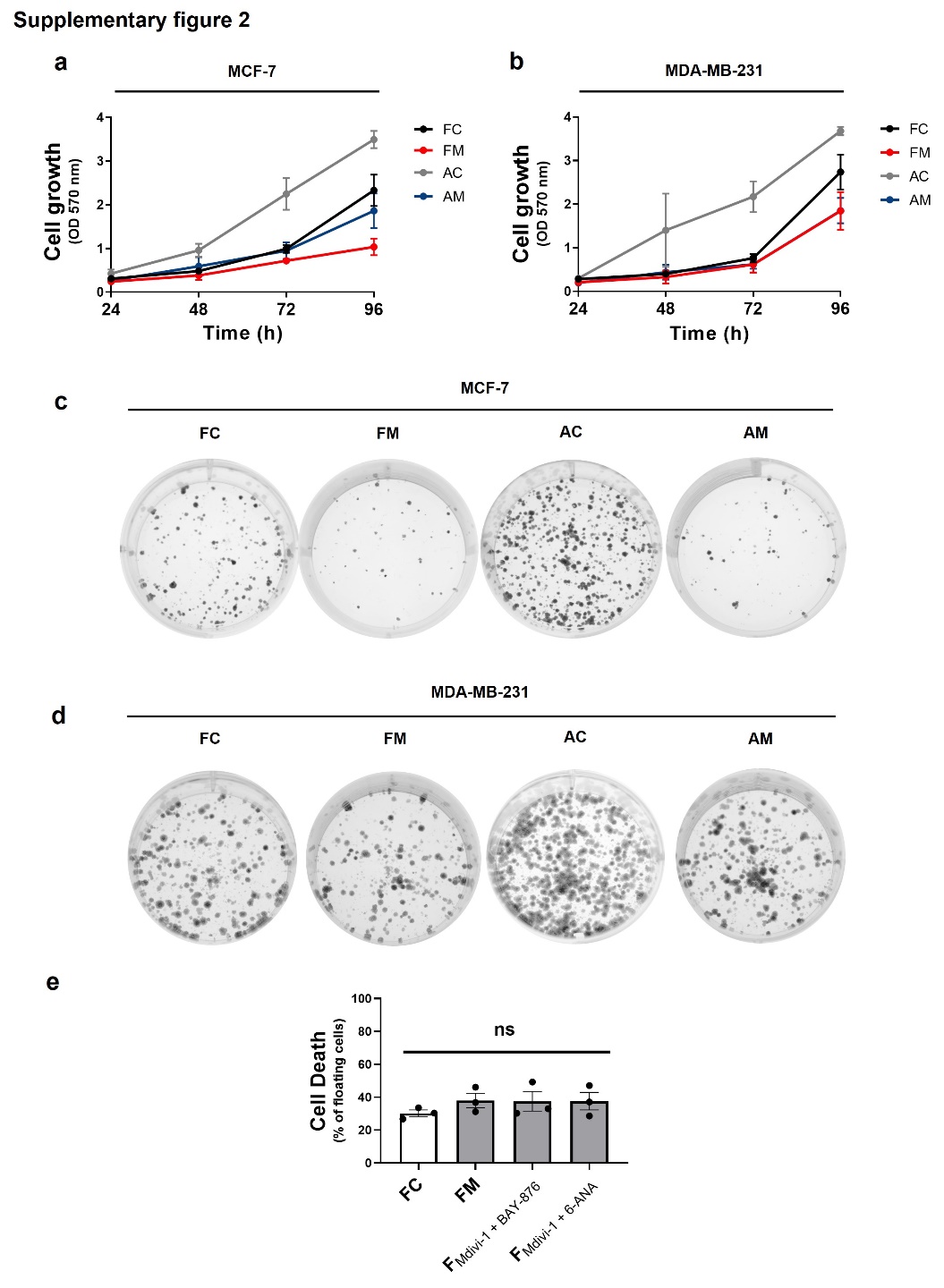


**Supplementary figure S2.** The proliferative and clonogenic potential upon re-seeding of the floating MDA-MB-231 and MCF-7 cells induced by mdivi-1. (**a)** MCF-7 and (**b**) MDA-MB-231 cells were treated with 50 μM mdivi-1 for 24 h. Floating and adherent cells were collected and re-seeded in an mdivi-1-free cell culture medium. Then, a proliferation assay by crystal violet was performed for 24, 48, 72, and 96 h. (**c**) MCF-7 and (**d**) MDA-MB-231 cells were treated with 50 μM mdivi-1 for 24 h. Next, cell populations were collected and re-seeded for the clonogenic assay. After seven days of growth, the colonies were photographed. (**e**) MDA-MB-231 cells were treated with 50 µM mdivi-1 plus 5 µM BAY-876 and 50 µM 6-ANA for 24 h. Then, floating control cells (FC), floating cells induced by mdivi-1 (FM), floating cells after treatment with mdivi-1 plus BAY-876 (FMdivi-1 + BAY-876), and floating cells after treatment with mdivi-1 plus 6-ANA (FMdivi-1 + 6-ANA) were collected, and cell death determine using SYTOX™ Blue dead cell stain and flow cytometry. Results represent means ± SEM. ns = not significant.


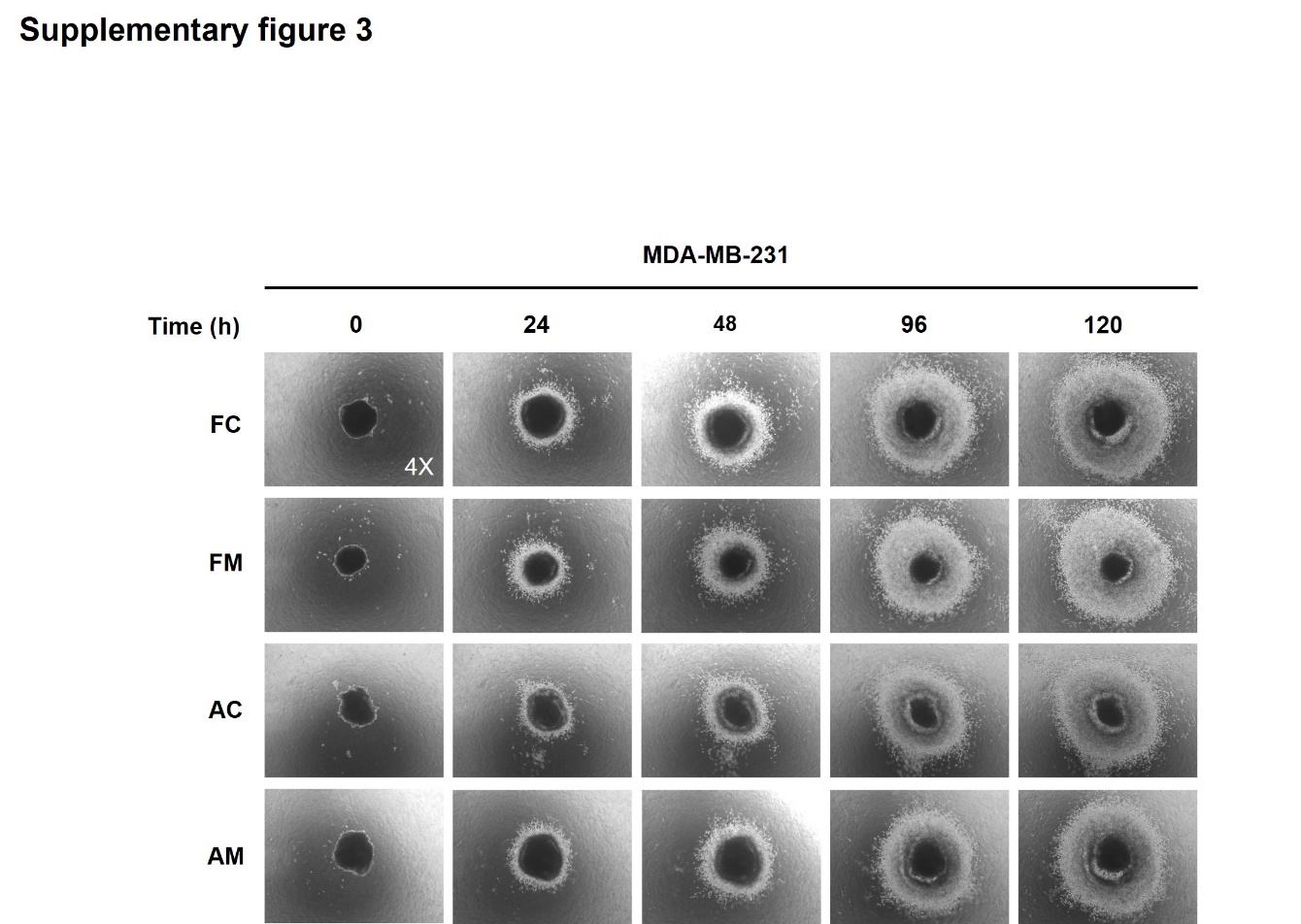


**Supplementary figure S3.** MDA-MB-231 cells were treated with 50 μM mdivi-1 for 24 h. Then, the four cell populations were collected to carry out the invasiveness assay for 24, 48, 96, and 120 h. These cells were photographed using a phase-contrast microscope.

**
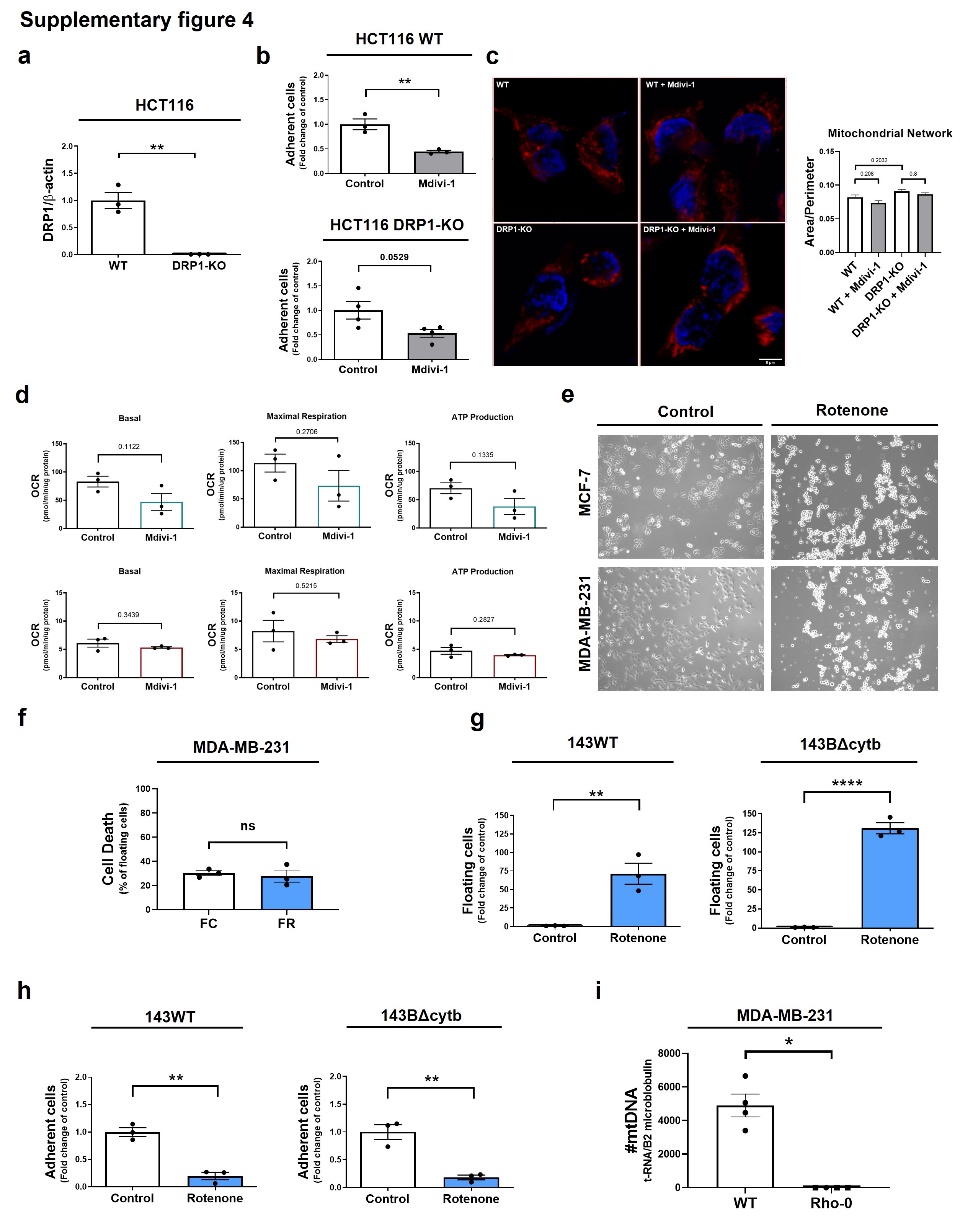
**

**Supplementary figure S4**. Mdivi-1 and rotenone induce cell detachment by a DRP1 and OXPHOS-independent mechanism. (**a**) Quantification of DRP1 and β-actin protein levels in HCT116-WT and KO DRP1 cells using ImageJ software**.** (**b**) HCT116**-**WT and KO for DRP1 cells were treated with 50 μM mdivi-1 for 24 h. The fraction of viable adherent cells was determined. (**c**) HCT116-WT and DRP1-KO cells were incubated with 50 µM mdivi-1 for 4 h. Then, HCT116 cells were treated with 100 nM MitoTracker™ Deep Red FM Dye. Images of the mitochondrial network were acquired using a Leica TCS SP8 Confocal Laser Scanning Microscope (Leica™). The scale bar corresponds to 5 µm. (**d**) Basal, maximal and ATP production link oxygen consumption rate (OCR) of MCF7 (upper panels) and MDA-MB-231 (bottom panels) cells incubated with 50 μM mdivi-1 for 4 h. (**e**) MCF-7 and MDA-MB-231 cells were treated with 10 µM rotenone for 24 h. These cells were photographed using a phase-contrast microscope. (**f**) MDA-MB-231 cells were treated with 10 µM rotenone for 24 h. Then, floating control cells (FC) and floating cells induced by rotenone (FR) were collected and cell death was determine using SYTOX™ Blue dead cell stain and flow cytometry. (**g, h**) 143Bwt and 143BΔcytb cells were treated with 10 μM rotenone for 24 h. The fraction of viable floating (**g**) and adherent (**h**) cells was determined. (**i**) Analysis of relative mitochondrial DNA (mtDNA) levels in MDA-MB-231 (WT) and Rho-0 cells. MtDNA copy number were determined using quantitative PCR (qPCR) by amplifying mtDNA (t-RNA) and nuclear DNA (B2 microglobulin). Results represent means ± SEM. *p< 0.05; **p< 0.01; ****p< 0.0001; ns = not significant.

**
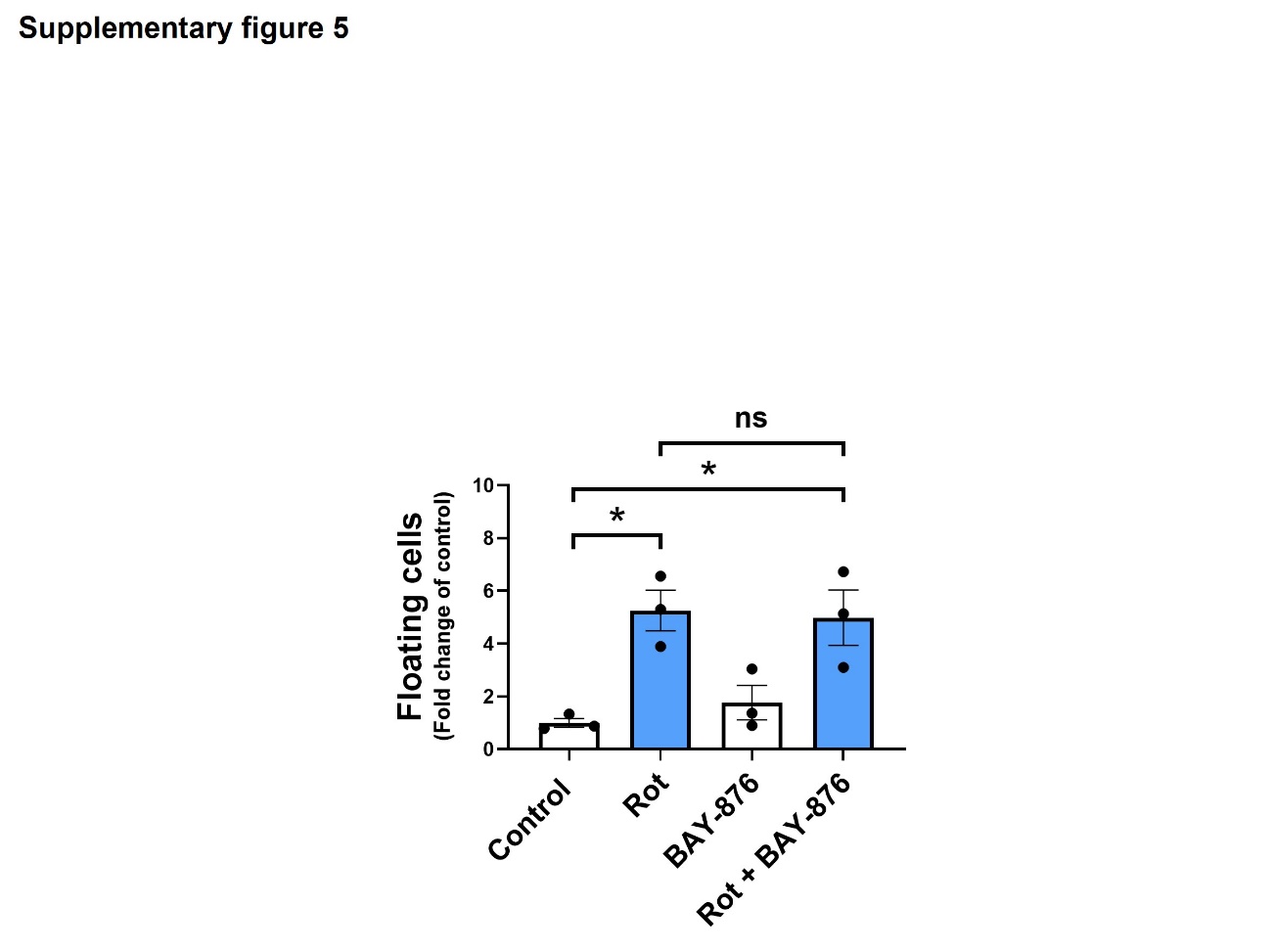
**

**Supplementary figure S5**. BAY-876 does not prevent the rotenone-induction of cell detachment of viable breast cancer cells. MDA-MB-231 cells were treated with 10 µM rotenone plus 5 µM BAY-876 for 24 h. Then, the fraction of viable floating cells was determined. Results represent means ± SEM. *p< 0.05; ns = not significant.

**
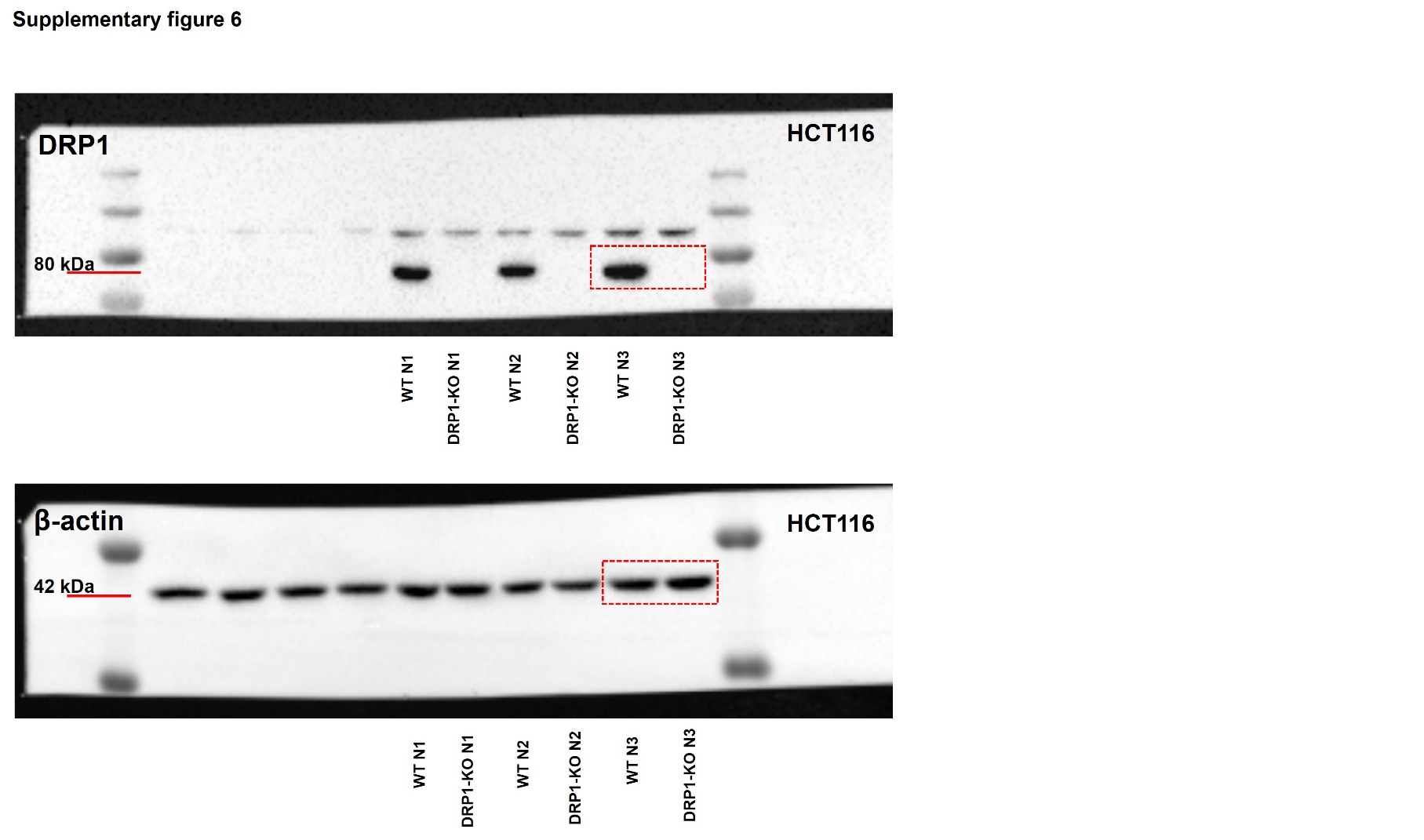
**

**Supplementary figure S6. Whole blot images of the DRP1 and β-actin protein levels in HCT116-WT and KO DRP1 cells. The DRP1 and β-actin images are from the same blot. The area outlined in red corresponds to the representative figure in Figure 4a.** For the **quantification of DRP1 and β-actin protein levels in HCT116-WT and KO DRP1 cells, we used N=3, as demonstrated in Supplementary Figure S4a.**
